# An Analysis of the Benefit of Using HEV Genotype 3 Antigens in Detecting Anti-HEV IgG in a European Population

**DOI:** 10.1371/journal.pone.0062980

**Published:** 2013-05-07

**Authors:** Annatina Schnegg, Philippe Bürgisser, Cyril André, Alain Kenfak-Foguena, Giorgia Canellini, Darius Moradpour, Florence Abravanel, Jacques Izopet, Matthias Cavassini, Katharine E. A. Darling

**Affiliations:** 1 Faculty of Biology and Medicine, University of Lausanne, Lausanne, Switzerland; 2 Services of Immunology and Allergy, Lausanne University Hospital, University of Lausanne, Lausanne, Switzerland; 3 Infectious Diseases, Lausanne University Hospital, University of Lausanne, Lausanne, Switzerland; 4 Service Régional Vaudois de Transfusion Sanguine, Epalinges, Switzerland; 5 Gastroenterology and Hepatology, Lausanne University Hospital, University of Lausanne, Lausanne, Switzerland; 6 INSERM, U1043, Centre de Physiopathologie de Toulouse Purpan, Toulouse, and CHU Toulouse, Hôpital Purpan, Laboratoire de Virologie, Institut Fédératif de Biologie de Purpan, Toulouse, France; Centers for Disease Control and Prevention, United States of America

## Abstract

**Background:**

The benefit of using serological assays based on HEV genotype 3 in industrialised settings is unclear. We compared the performance of serological kits based on antigens from different HEV genotypes.

**Methods:**

Taking 20 serum samples from patients in southwest France with acute HEV infection (positive PCR for HEV genotype 3) and 550 anonymised samples from blood donors in southwest Switzerland, we tested for anti-HEV IgG using three enzyme immunoassays (EIAs) (MP Diagnostics, Dia.Pro and Fortress) based on genotype 1 and 2 antigens, and one immunodot assay (Mikrogen Diagnostik recomLine HEV IgG/IgM) based on genotype 1 and 3 antigens.

**Results:**

All acute HEV samples and 124/550 blood donor samples were positive with ≥1 assay. Of PCR-confirmed patient samples, 45%, 65%, 95% and 55% were positive with MP Diagnostics, Dia.Pro, Fortress and *recom*Line, respectively. Of blood donor samples positive with ≥1 assay, 120/124 (97%), were positive with Fortress, 19/124 (15%) were positive with all EIAs and 51/124 (41%) were positive with *recom*Line. Of 11/20 patient samples positive with *recom*Line, stronger reactivity for HEV genotype 3 was observed in 1/11(9%), and equal reactivity for both genotypes in 5/11 (45.5%).

**Conclusions:**

Although *recom*Line contains HEV genotype 3, it has lower sensitivity than Fortress in acute HEV infection and fails to identify infection as being due to this genotype in approximately 45% of patients. In our single blood donor population, we observe wide variations in measured seroprevalence, from 4.2% to 21.8%, depending on the assay used.

## Introduction

Hepatitis E virus (HEV) is a single-stranded RNA virus acquired predominantly through faeco-oral transmission. Initially identified as a virus endemic in low-income regions, causing waterborne outbreaks of hepatitis, HEV is now recognised as the agent of a zoonotic infection, causing indigenous disease in industrialised countries [1].

Of the four HEV genotypes linked to human infection, outbreaks are generally caused by genotype 1 or 2, whilst genotype 3 is associated with autochthonous infection in humans, pigs and other mammals [2], [3], [4]. The clinical spectrum of acute hepatitis E in humans is broad, with asymptomatic infection in many cases [5].

Diagnosing HEV infection requires an understanding of the different phases of disease. In acute infection, HEV viraemia, as detected using PCR, is short-lived. Anti-HEV IgM and IgG are detectable at symptom onset, if symptoms occur. Thereafter, IgM titres fall over a period of weeks to months whilst IgG titres remain detectable for a period of one to several years [6], [7]. Most commercial enzyme immunoassays (EIAs) use antigens derived from HEV genotypes 1 and 2 [8]. The assays are based on proteins derived from two of the three open reading frames (ORFs) contained in the HEV genome, ORF2 and ORF3. ORF2 encodes the capsid protein and ORF3 a cytoskeleton-associated multifunctional protein. Assay sensitivity varies between different kits, for several reasons: assays for HEV antibodies based on recombinant proteins have been found to be more sensitive than those based on synthetic peptides [9]; antigenic properties of the epitopes, especially those of ORF2, are strongly conformation-dependent; and HEV sequence heterogeneity implies that antibody epitopes may not be conserved across strains. Differing assay sensitivities may partly explain the wide seroprevalence range reported in industrialised countries, from 0.2% to 52.5% [10], [11], [12]. Furthermore, the observation that certain serological tests have higher sensitivity for genotype 1 than for genotype 3 [2] suggests that anti-HEV IgG screening in industrialised countries, where indigenous infection is with genotype 3, is potentially hampered.

Given the recent introduction of diagnostic tests based on HEV genotypes 1 and 3, we compared the performance of an immunodot assay based on these two genotypes to that of three commercial EIAs based on genotypes 1 and 2 in two distinct populations from regions where HEV genotype 3 is the agent of autochthonous infection: 1) patients in southwest France in whom acute HEV infection due to genotype 3 had been diagnosed by real-time PCR and 2) asymptomatic blood donors (of unknown HEV status) in southwest Switzerland. The aims of this study were 1) to examine whether an assay based on genotype 3 would have superior sensitivity in a population of known HEV infection status and 2) to examine the range of seroprevalence measurements obtained by applying different assays to a single population.

## Materials and Methods

### Ethics Statement

Use of serum samples obtained from Toulouse, France, was part of a non-interventional study with no addition to the usual procedures. Biological material and clinical data were obtained only for standard viral diagnosis following physicians' orders (no specific sampling, no modification of the sampling protocol, no supplementary question to the national standardised questionnaire). Data analyses were carried out using an anonymised database. According to the French Law of Public Health (CSP Art L 1121–1.1), such protocol is exempt from written informed consent.

Use of blood donor samples from Lausanne, Switzerland, was approved by the Ethical Committee of the Canton of Vaud, Switzerland. All donors provided written consent to the use of blood samples in medical research.

### Sample Populations

Serum samples from patients living in the region of Toulouse, southwest France, with proven acute HEV infection, diagnosed on the basis of positive HEV RNA with concomitant clinical and biochemical evidence of acute hepatitis, were collected at the time of symptom onset (acute infection), or up to fourteen months after the acute phase (post-acute infection), and stored at −80°C. All HEV infections in these patients were identified as being due to HEV genotype 3, following real-time PCR based on ORF3 and ORF2 as previously described [13].

Samples from blood donors were collected consecutively and anonymously from 550 healthy blood donors living in the region of Lausanne, southwest Switzerland, in November 2009 as described previously [11]. Blood samples were stored at −80°C between the seroprevalence study performed in 2009 [11] and the present study. For analysis (see below), frozen samples were thawed in batches.

### Anti-HEV IgG EIAs

All samples were screened for anti-HEV IgG using three commercially available indirect EIAs: MP Diagnostics ELISA (MP Biomedicals SAS, Illkirch, France), Dia.Pro HEV IgG EIA (Dia.Pro Diagnostic Bioprobes Srl, Milan, Italy) and Fortress Diagnostics HEV-IgG EIA (Fortress Diagnostics Ltd, Antrim, UK). The MP Diagnostics (formerly Genelabs Diagnostics, Singapore) kit is an indirect EIA using three recombinant fusion proteins: one containing a 42-amino acid sequence from the ORF2 of the Mexican strain (genotype 2), one containing a 33-amino acid sequence from the ORF3 of the same Mexican strain, and one containing the homologous ORF3 sequence from the Burmese strain (genotype 1) [14], [15]. According to the manufacturer, sensitivity and specificity using this kit are 97% and 98%, respectively. The Dia.Pro kit is sold in France under the name Adaltis by InGen (InGen France, personal communication). The assay uses four synthetic peptides representing epitopes from ORF2 and ORF3 from the Burmese and Mexican HEV strains. These peptides have the same C-terminal amino acid as the recombinant sequences present in the MP Diagnostics test but differ at the N terminus, being shorter by 10 residues (ORF3) and longer by 46 residues (ORF2). According to the manufacturer, sensitivity and specificity reach 100%. The Fortress Diagnostics kit is identical to that produced by Wantai (Beijing Wantai Biological Pharmacy Enterprise Co., Ltd, China) [16] and uses one long recombinant protein (PE2) containing 211 amino acids of the ORF2 of a Chinese strain belonging to genotype 1. This protein forms homodimers and polymers that have greatly enhanced antigenicity compared to the monomeric form [16], [17], [18]. Specificity and sensitivity are not indicated by the manufacturer. The three EIAs were each performed according to the manufacturers’ instructions. In our analyses, any sample with an initial optical density (OD)/cut off ratio of ≥0.9 was retested in duplicate and was considered positive if the OD/cut off ratio of both replicates was ≥1.0.

### Anti-HEV IgG Immunodot Assay

Samples testing positive with at least one EIA underwent further testing using a line immunodot assay, Mikrogen Diagnostik *recom*Line HEV IgG/IgM (Mikrogen GmbH, Neuried, Germany). This assay uses four recombinant proteins applied separately to a nitrocellulose strip: O2 N, O2 M and O2C, which represent approximately the N-terminal, middle and C-terminal third of the ORF2 protein, respectively, and O3, which consists of the full-length ORF3 protein. Each O2 N, O2C and O3 are present as two bands, one with a genotype 1 sequence, the other with a genotype 3 sequence; for O2 M, only a genotype 1 sequence is provided. After sequential incubation with each sample, an anti-IgG conjugate and a chromogenic substrate, the nitrocellulose strips are scanned using a Plustek OpticPro S28 apparatus and the results are evaluated using the *recom*Scan program of Mikrogen according to the manufacturer's instructions. Briefly, each individual HEV band on the strip is rated where the sensitivity cut off is defined by the intensity of a control band. Any HEV band undetectable (-), or with an intensity weaker than that of the control band (±), is rated negative. Any HEV band with an intensity equal to (+) or stronger than (++, +++) the control band intensity is rated positive. If the intensity of two homologous bands (one from genotype 1 and one from genotype 3) differ, only the stronger band is rated. Second, the whole strip is rated, where each positive HEV band, whatever its intensity (+,++or +++), is attributed a number of points according to the protein identity (O2 N: 2 points, O2C: 4 points, O2 M: 2 points, O3∶3 points). The sum of the points for the four non-homologous bands (the brighter one of the two genotypes) of each strip is then calculated. The final result may be negative (sum ≤2), borderline (sum = 3), or positive (sum ≥4). According to the manufacturer, sensitivity is 100% and specificity is 98.8%. This assay differs from a previously described immunoblot assay called RecomBlot [2], also produced by Mikrogen, which is based on HEV genotypes 1 and 2.

For the 20 serum samples from patients with HEV RNA-proven HEV infection, we calculated sensitivity as the percentage of samples testing positive with each test. For the 550 blood donor samples, we examined the percentage of samples testing positive with each test and expressed this as the ‘measured seroprevalence’ for the given assay.

### Data Analysis

Data are expressed as percentages to denote sensitivity and seroprevalence, according to the sample population described. Correlation was measured by calculating Spearman’s rank correlation coefficient. All analyses were performed using Microsoft Excel 2008 (Microsoft Corporation, Redmond, WA).

## Results

### Study Population

The patient population comprised 15 patients (12 men, three women) presenting with clinical and biochemical features of acute HEV infection, and five patients (three men, two women) with post-acute infection, presenting 4–14 months after the acute phase. Median patient age was 50.5 years (interquartile range 43–60 years). Measured ALT values were available in 7/15 patients with acute HEV (mean 1631±843 IU/L) and in 2/5 patients with post-acute infection (mean 53±12 IU/L). The blood donor population comprised 332 men and 218 women, median age 55 years (interquartile range 46–63 years); 99.3% had normal ALT values. In this group, none was positive for HIV or hepatitis B surface antigen; one individual had antibodies to hepatitis C virus (HCV) but was HCV RNA negative.

Of the patient samples from the acute phase of HEV infection, 8/15 (53%) were positive with MP Diagnostics, 12/15 (80%) were positive with Dia.Pro, 14/15 (93%) were positive with Fortress, and 6/15 (40%) were positive with *recom*Line (Table 1). Of the post-acute samples, 1/5 (20%) were positive with MP Diagnostics and Dia.Pro, and 5/5 (100%) were positive with Fortress and *recom*Line. Of the six acute samples positive with *recom*Line, five showed stronger reactivity for genotype 1 than for genotype 3, and one showed equal reactivity for both genotypes; of the five post-acute samples, four had equal reactivity for both genotypes and one showed greater reactivity for genotype 3 (Table 1). Examining test performances with all patient sera, we observed a correlation between the strength of reaction with all three EIAs and the score with the *recom*Line test (r = 0.63 for Dia.Pro, *P* = 0.007 to r = 0.8 with MP Diagnostics, *P* = 0.001).

**Table 1:** Results of patient serum samples using the different anti-HEV IgG assays.

Patient Sample	MP Diagnostics (Genelabs)		Dia.Pro		Fortress		Mikrogen dot		
	Mean result	Interpretation	Mean result	Interpretation	Mean result	Interpretation	Score	Interpretation	Genotype
1	1.4	Pos	3.67	Pos	0.4	Neg	0	Neg	–
2	0.4	Neg	2.93	Pos	1.09	Pos	0	Neg	–
3	6.28	Pos	9.1	Pos	13.43	Pos	3	BL	–
4	6.15	Pos	9.3	Pos	18.1	Pos	11	Pos	1>3
5	0.1	Neg	2.37	Pos	3.93	Pos	0	Neg	–
6	0	Neg	0.3	Neg	7.21	Pos	0	Neg	–
7	1.11	Pos	5.23	Pos	15.03	Pos	4	Pos	1>3
8	4.94	Pos	8.3	Pos	4.23	Pos	7	Pos	1>3
9	0.2	Neg	0.97	Neg	4.92	Pos	0	Neg	–
10	6.67	Pos	9.17	Pos	17.82	Pos	9	Pos	1 = 3
11	0.8	Neg	9.3	Pos	10.25	Pos	0	Neg	–
12	6.17	Pos	9.83	Pos	13.21	Pos	9	Pos	1>3
13	0.1	Neg	0.2	Neg	12.84	Pos	0	Neg	–
14	5.3	Pos	8.53	Pos	17.92	Pos	7	Pos	1>3
15	0.3	Neg	2.53	Pos	5.97	Pos	0	Neg	–
16	0.1	Neg	0.1	Neg	17.23	Pos	4	Pos	1 = 3
17	0.1	Neg	0.1	Neg	15.64	Pos	4	Pos	3>1
18	0.3	Neg	0.8	Neg	18.04	Pos	4	Pos	1 = 3
19	0	Neg	0.3	Neg	18.07	Pos	4	Pos	1 = 3
20	6.75	Pos	9.23	Pos	17.94	Pos	7	Pos	1 = 3

Samples 1 to 15 were obtained from patients with documented HEV infection (positive HEV PCR); samples 16 to 20 were obtained from patients 4–14 months following the acute phase.

Of 550 blood donor samples, 124/550 (22.5%) tested positive with at least one EIA kit: 27/550 (4.9%) were positive with MP Diagnostics, 23/550 (4.2%) were positive with Dia.Pro, and 120/550 (21.8%) were positive with Fortress. Of these positive samples, 51/124 (41%) were positive, 67/124 (54%) were negative, and 6/124 (4.8%) were borderline with the *recom*Line kit (Table 2).

**Table 2:** Breakdown of all donor samples positive with each enzyme immunoassay (EIA) kit and results of the *recom*Line immunodot.

EIA test result combinations				Results with *recom*Line		
MP Diagnostics	Dia.Pro	Fortress	Total for each combination	Negative	Borderline	Positive
Pos	Pos	Pos	19	1	2	16
Pos	Neg	Pos	4	1	1	2
Neg	Pos	Pos	3	1	0	2
Pos	Pos	Neg	1	1	0	0
Neg	Neg	Pos	94	61	2	31
Pos	Neg	Neg	3	2	1	0
Neg	Pos	Neg	0	0	0	0
Total			124	67	6	51

The Dia.Pro and the MP Diagnostics kits had concordant results for 540/550 (98.2%) samples, 20 (3.6%) being positive and 520 (94.5%) being negative with both tests. Of the 20 double positive samples, 16 were also positive with the *recom*Line test. The Fortress test yielded the highest proportion of positive results 120/550 (21.8%). However, fewer than half of these (51/550, 9.3%) tested positive with the *recom*Line test. Of the 430 samples testing negative with the Fortress kit, three tested positive with the MP Diagnostics kit and one tested positive with both MP diagnostics and Dia.Pro kits. Of these four samples, none yielded a positive result with the *recom*Line test.

Of the samples positive with ≥1 EIA and with *recom*Line, the majority (44/51, 86%) scored 4 points (intensity greater than the control band for O2C only), with the remainder (7/51, 14%) scoring 7 points (intensity greater than the control band for proteins O2C and O3); all *recom*Line-positive samples were either undetectable or with an intensity weaker than that of the control band for proteins O2 M and O2 N. Considering HEV genotypes in positive *recom*Line samples, the intensities of the homologous bands were equal for both genotypes (genotypes 1 and 3) in 25/51 samples (49%) and stronger for genotype 3 in 26/51 samples (51%).

**Figure 1:**
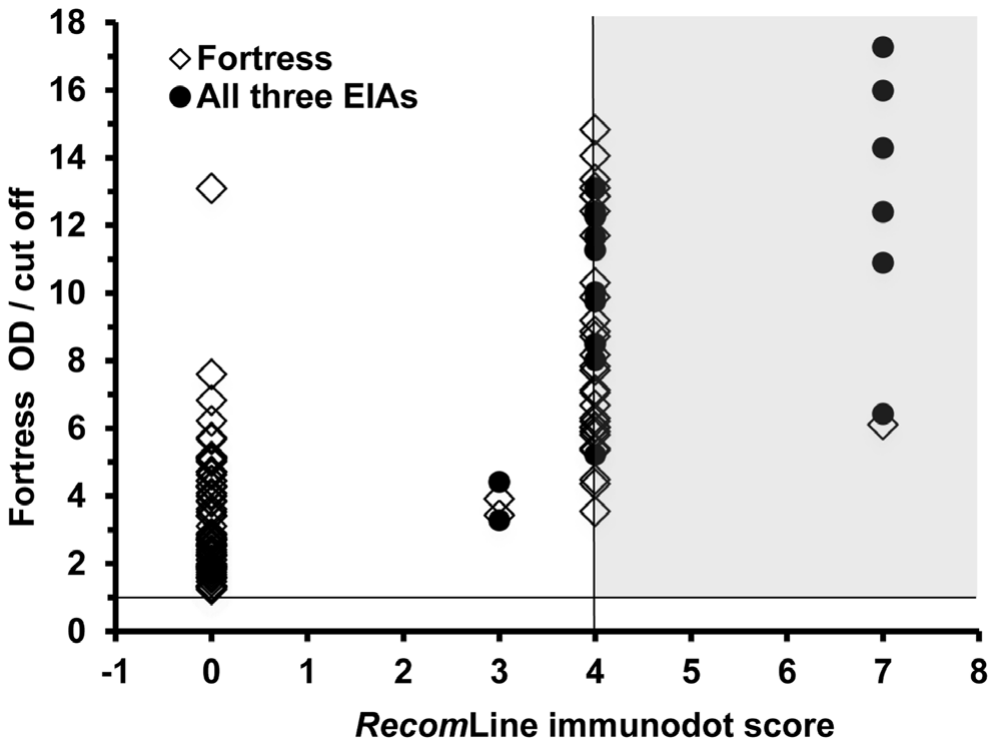
Correlation between strength of reaction and *recom*Line score. *Recom*Line immunodot score and optical density (OD)/cut off ratio for samples positive only with the Fortress kit and those positive with all three EIAs. The shaded zone demarcates samples which were positive both with Fortress (according to our study protocol, where an OD/cut off ratio ≥1.0 is considered positive) and with *recom*Line.

Examining all the donor samples, we observed a correlation between the Fortress test reaction strength and the *recom*Line score (r = 0.74, *P* = 0.0001) (Figure 1). However, for the Dia.Pro (r = 0.479, *P* = 0.05) and the MP Diagnostics tests (r = 0.345, *P* = 0.12), this correlation was less strong.

## Discussion

We have examined the performance of three EIAs and one immunodot assay in two distinct populations: a patient population with acute/post-acute HEV infection and an asymptomatic blood donor population. In the patient population, we were able to determine assay sensitivity as all samples came from individuals with PCR-proven HEV infection. We found the Fortress EIA to have the highest sensitivity: 95% in all patients and 100% in the post-acute phase subgroup (five patients). The *recom*Line immunodot, despite being based on HEV genotypes 1 and 3, and despite being applied to samples with proven genotype 3 infection, had an overall sensitivity of 55%. Although this improved to 100% in the post-acute subgroup, identification of infection as being due specifically to genotype 3 was achieved in a single patient. In the blood donor population, in whom HEV status was unknown, we observed marked differences in measured HEV seroprevalence, from 4.2% to 21.8%, depending on the assay used.

Commercial assays for detecting anti-HEV IgG are required in two main settings: 1) as a diagnostic test and 2) to measure HEV seroprevalence in a given population. Compared to assays for anti-HEV IgM, with which non-specific reactions are described [6], non-specific reactivity with anti-HEV IgG is encountered less frequently. As such, different EIA results are more likely to occur from differences in sensitivity than specificity. Several explanations exist for the differing kit performances we observe. The kits we used are not based on the same antigen length and use different genetic sequences, expressed in different systems: the MP Diagnostics and Fortress kits use recombinant proteins while the Dia.Pro kit is based on synthetic peptides. Second, the presence of conformational epitopes in the long ORF2 sequence provided in the Fortress kit is likely to play a major role; given that all the *recom*Line-positive samples were O2C-reactive, it is possible that the O2C band recognises epitopes which are present on PE2 (Fortress) but not on the short peptides of the other two EIAs.

Taking the Fortress and *recom*Line figures, as both these tests had high sensitivity in the post-acute subgroup of patients, there are two possibilities to explain the differences in measured seroprevalence values: 1) that *recom*Line has too low a sensitivity to detect antibodies at low titres; 2) that Fortress lacks specificity in this non-epidemic population. Given that HEV infection is often asymptomatic, it is difficult to prove that a negative screening test means absence of previous infection. The best argument that Fortress does not lack specificity comes from seroprevalence data in southwest France among 188 children 2 to 4 years old (3.7%) against a figure of 52.5% in adults in the same region [12]. The low sensitivity of the *recom*Line assay as compared to Fortress can also be seen in the patients with acute infection, when IgG antibodies are still rising in concentration and, perhaps more importantly, are still of low avidity.

Considering the *recom*Line immunodot as a screening test, this assay uses the specific antigen ORF3 [2] and three ORF2 antigens, of which only one (O2C) is sufficient to give a positive result on its own. Of the six blood donor samples with reactivity by *recom*Line limited to O3, and so borderline, five were weakly reactive with Fortress. These samples might be false positives with Fortress or true positives due to higher antigenicity of PE2 over O2C. However, given the correlation we observe between reaction strength (OD/cut off ratio) of the Fortress assay and the *recom*Line score, further studies are needed to investigate whether anti-O3-alone may be indicative of past infection.

The low sensitivities we observe for the MP Diagnostics and Dia.Pro kits are surprising, given the manufacturers’ own figures. However, our observations are in keeping with those of Bendall and co-workers who observed that the MP Diagnostics kit, commercialised under the name of Genelabs HEV IgG EIA (Genelabs, Inc., Singapore), was more sensitive early in HEV infection than after the acute phase [16]; in a sample population fulfilling criteria for blood donation, any individuals positive for anti-HEV IgG are likely to be in the non-acute phase. When screening individuals in the non-outbreak or low prevalence context, an assay of high sensitivity is required and we propose that the Fortress EIA fits this profile. The exception is chronic hepatitis E in immunocompromised individuals in whom anti-HEV IgG may be negative and in whom the diagnosis should be made by PCR for HEV RNA [19], [20].

This study has limitations. The main limitation is that, while we were able to measure assay sensitivity in the patient population, taking PCR as a gold standard test for HEV infection, we have no gold standard test to apply to the population of blood donors. By definition, asymptomatic blood donors have no symptoms of acute or recent HEV infection and so, even if they have been infected with HEV, they are beyond the window of PCR-demonstrable viraemia. In the patient population, the samples in which to determine each assay’s potential for measuring seroprevalence were those from the patients with post-acute infection. However, these samples were few in number: five patients of 20 with PCR-proven infection. To address the question of sensitivity in measuring seroprevalence, we would need to take PCR-proven cases and follow them longitudinally to examine the performance of different assays at different time points. This would still leave the problem of determining specificity: as HEV infection may be subclinical, it is not possible to identify truly negative individuals who could serve as controls. Second, with respect to our seroprevalence figures, the mean age of our blood donor population was 55 years old, as donors were recruited from centres other than university campuses and the military, as previously described [11]; as HEV prevalence has been observed to increase with age [11], [21], the findings in our population may overestimate HEV seroprevalence in Switzerland as a whole. Against this, the aim of this study was to examine the benefit of using an anti-HEV IgG assay based on HEV genotype 3 antigens in populations in which this genotype is the cause of indigenous infection, rather than specifically to measure seroprevalence.

In summary, we have observed highly variable performances in both the acute setting and in the measurement of seroprevalence between currently available commercial tests detecting anti-HEV IgG. Our results suggest that epidemiological studies not using identical screening assays should not be compared as observed differences between populations may be explained by differences in assay sensitivity as well as by true differences in seroprevalence. Finally, our data show no benefit in using a screening assay based on HEV genotype 3 antigens, either in demonstrating infection or in identifying the responsible genotype, even in populations from industrialised regions.
